# Nature connectedness framework for adolescents: an integrative review

**DOI:** 10.3389/fpsyg.2026.1721907

**Published:** 2026-03-04

**Authors:** Gulsah Yildirim, John Willison, Brendan Bentley

**Affiliations:** School of Education, College of Education, Behavioural and Social Sciences, Adelaide University, Adelaide, SA, Australia

**Keywords:** adolescents, conceptual framework, human-nature connection, integrative review, nature connectedness

## Abstract

Connection to nature has been recognized to be critical for health, wellbeing and eco-supportive actions. While measures of children’s connection to nature are relatively high, studies indicate a decline during early adolescence. Inconsistencies in existing research on conceptualization and measurement, along with the need to consider the unique experiences and perspectives of younger generations, highlight the need for an improved conceptual framework that guides the development of instruments that assess connection to nature. This article presents an integrative review that synthesized empirical literature from diverse fields to identify key dimensions of nature connectedness: emotional and relational connection, cognitive connection, experiential and sensory engagement, philosophical-ethical-spiritual connection, sustainable practices and stewardship, and psychological and physical wellbeing. An iterative integrative review methodology involving concept mapping and peer review feedback was employed to develop the Integrated Nature Connectedness Framework. The resulting framework is expected to guide the development of age-appropriate instruments for assessing connection to nature. By providing a holistic understanding of human-nature relationships in the context of a rapidly changing, technology-driven world, the Integrated Nature Connectedness Framework aims to inform educational strategies that effectively nurture nature connectedness and sustainable behaviors of adolescents.

## 1 Introduction

### 1.1 Significance of nature connectedness

In an increasingly changing world driven by urbanization and technology, connection to nature has proven to be important for human wellbeing and ecological understanding has emerged as a powerful motivator for pro-environmental action. Addressing the challenge of urbanization, technology and ecological sustainability is essential for long-term health of the environment and supporting the delicate balance between human interaction and the natural world. Enhancing connection to nature, especially the connectedness of young people, may be one of the most effective ways of addressing these challenges.

Research suggest that the natural world is facing an increasing number of environmental stressors (Zhou et al., 2024). The degradation of the environment, including a decrease in biodiversity, the fragile balance of ecosystems globally and widespread pollution, is evidence of the destruction of natural habitats (IPBES, 2019). The depletion of natural resources, fueled by unsustainable consumption patterns and rapid population growth, add to the immense strain on the Earth’s finite resources (Huo and Peng, 2023). Amidst these interrelated issues, researchers have highlighted that cultivating a deep connection to nature has become a crucial strategy for inspiring pro-environmental behaviors (Whitburn et al., 2020; Lovati et al., 2023). The specific connection between humans and nature has become increasingly popular subject of interest and research. Understanding the role of connecting to nature is important in fostering mutually beneficial and sustainable relationships with people and the natural environment.

Connection to nature has frequently been associated with individual physical, mental and social health, which are integral to overall wellbeing (Charles and Louv, 2009; Kovi et al., 2023). However, the link between wellbeing benefits and connection to nature cannot be explained by this association alone. A substantial body of research shows that exposure to natural environments, even in passive forms, is associated with reduced stress and improved wellbeing. Stress Reduction Theory (SRT) suggests that stress levels reduce significantly in natural settings because of human innate preference for nature which stems from human evolutionary history (Ulrich et al., 1991), even though human interaction with nature could be cast as survival-based and potentially stressful. Such accounts help explain why contact with nature supports wellbeing, yet the accounts should not be interpreted as implying that connection to nature emerges automatically from exposure. Rather, contemporary conceptualizations of connection to nature emphasize a broader bond with the natural world, which has been associated with common wellbeing constructs such as life satisfaction, vitality, and happiness (Capaldi et al., 2014). A strong bond with the natural world is associated with hedonic wellbeing through the provision of pleasure and reduction of stress, and eudemonic wellbeing through fostering a sense of purpose, meaning, and belonging (Pritchard et al., 2020). Connection to nature has a mediating effect on the associations between nature exposure and a range of outcomes, including blood pressure, cortisol levels, brain activity, cognitive performance and overall mental health (Gal and Domotor, 2023). In contrast, the lack of connection to nature, often referred to as “nature deficit disorder,” has been linked to various mental and physical health problems (Charles and Louv, 2009).

Studies have also highlighted the impact that the human connection to nature has on environmental sustainability (Ives et al., 2017; Whitburn et al., 2020). Connecting with nature is a crucial element in shaping attitudes and behaviors toward environmental protection through a subjective sense of the relationship with nature (Baird et al., 2022; Lovati et al., 2023; Whitburn et al., 2020). Recent literature reviews assert that this relationship mediates engagement with nature (Gal and Domotor, 2023) and the promotion of pro-environmental conduct (Lovati et al., 2023) such as the adoption of more sustainable lifestyles and actions toward environmental protection (Barros and Pinheiro, 2020). However, stronger connection to nature does not always align with greater intentions to act pro-environmentally (Baird et al., 2022) and therefore requires further research.

Within the literature, several related terms are frequently used fungibly, with substantial overlap in how they are operationalized across studies. To strengthen conceptual clarity in the present review, we distinguish between connection to nature, contact/exposure, interaction with nature, and environmental engagement, articulating these as distinct constructs. Throughout the paper, connection to nature refers to a dynamic psychological-relational construct including emotional bonding and understanding the interconnectedness with the natural world (Mayer and Frantz, 2004; Nisbet et al., 2009). In contrast, contact or exposure to nature refers when people are surrounded by a natural environment or encounter natural elements (Garza-Teran et al., 2022). Interaction with nature, by comparison, is used more narrowly to describe having a direct contact with nature from a sensory perspective rather than mere presence or passive exposure (Duke and Holt, 2023) which is more specific and might refer to the activities or experiences in natural places. Environmental engagement refers to behaviors oriented toward environmental care or protection for sustainability (Manoj M. et al., 2020). In this review, engagement with nature is used synonymously with interaction with nature, referring to active experiences in natural settings. The above clarification enables a clearer synthesis of the factors that shape nature connectedness across contexts.

Although a body of research has investigated the topic of human connection to nature, several key areas remain unresolved. Identifying the key influences on nature connectedness is integral for understanding and assessing the concept.

### 1.2 Adolescence as a distinct developmental phase

Connection to nature is considered a dynamic construct that evolves throughout a person’s life (Nisbet et al., 2009; Mayer et al., 2009). This evolving relationship with the natural world is shaped by experiences and by dimensions of personal identity, including dispositional traits and cultural orientations (Richardson et al., 2019). Importantly, adolescence represents a distinct developmental phase in which nature connectedness may be reshaped by substantial cognitive, emotional, and social changes, which makes it sensitive to contemporary contextual influences. In recent decades the human-nature relationship has undergone particularly rapid transformation. Researchers have suggested these changes may have been as a result of various factors including the rapid change in technological innovation, variations and growing differences in the understandings across children, adolescents and adults, and the influence of educational environments.

#### 1.2.1 Technology-mediated engagement with nature

Several researchers support the idea of the impact technology has in affecting the human-nature relationship (Calogiuri et al., 2023; Brambilla et al., 2024). Technology and its rapid change are considered to impact nature connectedness, shaping individual experiences, social relationships, and perception. Research has demonstrated that technological advancement has been associated with both facilitative and inhibitive effects on human-nature relationships (Litleskare et al., 2020). Contemporary digital technologies, including mobile devices, interactive platforms, and immersive media systems, have enhanced accessibility to nature-simulative experiences and environmental education resources, particularly for populations with limited direct access to natural environments (Brambilla et al., 2024). Several emerging technologies in particular such as Artificial Intelligence (AI), Augmented Reality (AR), and Virtual Reality (VR) have been explored for their potential to support nature connectedness through personalized environmental interventions. These technologies demonstrate effectiveness through their ability to overcome geographical and physical barriers, create safe and standardized conditions for exploration, and offer multisensory immersion that is often not available in unstructured outdoor contexts (Spangenberger et al., 2024; Crogman et al., 2025). Supporting this positive perspective, studies assert that nature connectedness can be enhanced through a short walk in a virtual natural environment (Calogiuri et al., 2023) or augmented storying about nature in school classes (Kumpulainen et al., 2020). Yet findings remain mixed. Other research presents no significant difference, suggesting that digital technologies may not consistently facilitate nature connectedness across all implementation contexts (Sneed et al., 2021; Spangenberger et al., 2022).

Although digital technologies can be leveraged to nurture the human-nature relationship, this effect is not linear. Experts caution that technology overuse already limits younger generations’ outdoors time, and VR nature experiences may further replace actual interaction with nature (Sultana and Hawken, 2023). While immersive virtual nature experiences may offer certain advantages such as fostering wellbeing (Sneed et al., 2021), it is significant to recognize their limitations in fully replicating the comprehensive, multi-sensory, and potentially exhilarating encounters with real nature (Litleskare et al., 2020). Direct contact with the natural places offers unique advantages, such as improved immunity through acquiring microbiomes from the ecosystem (Kuo, 2015), which are beyond the scope of current technologies. Moreover, risks such as cliff lines and strong sun provoke different character-building responses. Despite future technological advancements, virtual nature may never entirely capture the authenticity of genuine nature experiences. Technology could serve as a valuable middle ground to bridge the growing disconnect between people and natural environments (Litleskare et al., 2020) or it could further exacerbate this gap; indeed, digital technologies have already contributed to declines in face-to-face interaction and family engagement, particularly among young people (Buecker et al., 2021). This decline has weakened social ties between friends and family networks, where values and orientations toward nature and the environment are often transmitted and reinforced (Buecker et al., 2021; Oh et al., 2021). Weakening social ties may then detract from the development of connection to nature, however this is not necessarily a unidirectional force, as such weakening may also contribute to the tendency of people to connect to nature in a human-centered or anthropomorphic way, focusing more on species perceived as cute, mammals, or “smart” (Schwartz and Beran, 2022). Consistent with this, Epley et al. (2008) state that when people feel socially disconnected, they tend to connect more with their pets, often attributing them with human-like qualities such as being caring or thoughtful companions. It is possible that such compensatory tendencies also extend to a broader sense of connection with the natural world (Myers and Saunders, 2002). Empirical evidence for this extension, however, remains limited.

#### 1.2.2 Generational understandings

Further evidence of change in the human-nature relationship is reflected in the variations and growing differences in the understandings between age groups. Differences have been identified in the level of connection that children and adults have in their relationship with the natural world. Studies consistently demonstrate that children exhibit higher levels of connection to nature when compared to adults (Chawla, 2020; Price et al., 2022). This connection tends to decline as children grow older, with a particularly sharp decrease observed during adolescence (Keith et al., 2021; Bezeljak et al., 2023). This phenomenon, often referred to as the ‘teenage dip,’ is most pronounced after the age of 11–12 years (Richardson et al., 2019; Price et al., 2022). Keith et al. (2021) suggest that the age when children’s connection to the natural world begins to dramatically decrease is precisely between 11 and 12 years-of-age. This age range distinguishes two distinct cohorts that align with primary school students having a relatively high connection to nature and secondary school students being relatively disconnected from nature (Keith et al., 2021). Despite this decline in nature connectedness, adolescents still frequently mention nature as their favorite place (Price et al., 2022). Adolescents are involved in a progression of stages requiring that they deal with physical development, acceptability by the group, love, and job decisions. Their shifting focus and priorities on personal and social identity might result in weakened connection with nature during this critical developmental stage (Richardson et al., 2019).

#### 1.2.3 Educational environments

A number of studies support the idea that schools are significant agents in the fostering of adolescent nature connectedness. These educational institutions provide opportunities for immersive nature learning, emphasizing ecology, sustainability, and human-nature relationships within curriculum. Vella-Brodrick and Gilowska (2022) suggest schools play a crucial role in nurturing their identity development of children and adolescents who spend a substantial portion of time in the classroom. Furthermore, educators have the opportunity to assist them in shaping new ideas and opportunities regarding their relationship with nature (Vella-Brodrick and Gilowska, 2022). However, the human-nature relationship is complex and includes a wide range of experiences, interactions, and contexts, and research investigating the impact of education programs rooted in nature on children’s connections reveals conflicting outcomes. While some researchers have stated that after sustainability education programs, children evidence heightened nature connectedness for weeks (Braun and Dierkes, 2017), other research has found no long-term impacts or that initial increases are lost and there is a return to baseline levels within weeks (Sellmann and Bogner, 2013).

### 1.3 Conceptualizations and measurement of connection to nature

Nature connectedness has long been a central focus in environmental psychology and education research (Beery et al., 2024). A number of scales measuring the relationship between human and nature have been developed. Early concepts of the human-nature relationship emphasized the emotional affinity to nature. The measure developed by Kals et al. (1999) assessed emotional tendency for the natural world by the Emotional Affinity toward Nature (EATN) scale. Concepts that focus on environmental identity position connection to nature as a component of self-concept. Schultz (2001) Inclusion of Nature in Self (INS) scale and Clayton (2003) Environmental Identity (EID) scale assessed the extent to which nature is integrated into one’s self-concept. Later, more comprehensive models emerged. Mayer and Frantz (2004) Connectedness to Nature Scale (CNS) operationalize connection to nature as an emotional bond with the natural world. However, subsequent analyses questioned its construct validity, noting that although it was intended to capture affective connection, many items instead reflect cognitive appraisals rather than genuine emotional experiences (Perrin and Benassi, 2009). This raised concerns that the CNS may not adequately capture the emotional dimension of human-nature relationships.

Recognizing this limitation, researchers developed measures that more explicitly targeted affective aspect. Perkins (2010) the Love and Care for Nature (LCN) scale and Cheng and Monroe (2012) Connection to Nature Index (CNI) were designed to capture emotional affinity to nature. Tam (2013) Dispositional Empathy with Nature (DEN) further extended the line of work and highlighted empathy as a distinct dimension of connectedness, particularly in response to ecological threat. These advances reflected growing recognition that affective bonds constitute a distinct and influential aspect of connectedness.

At the same time, broader multidimensional psychological instruments emerged. The Connectivity with Nature (CWN) scale (Dutcher et al., 2007) measures affective and experiential connections to nature, while the Nature Relatedness (NR) scale (Nisbet et al., 2009) also assesses cognitive aspects. The Commitment to Nature (COM) scale (Davis et al., 2009) comprises behavioral dimension, measuring willingness to invest resources into nature-related activities. Comparative analyses suggest that these multidimensional measures are highly correlated and converge on the same construct (Tam, 2013) which is connection to nature. While these scales directly operationalize connection to nature, other instruments have been developed to capture broader orientations toward the environment. The New Ecological Paradigm (NEP) scale (Dunlap et al., 2000) has been used to assess environmental attitudes and beliefs, while the Disposition to Connect with Nature (DCN; Brügger et al., 2011) aims to capture the general tendency toward the natural world.

More recently, efforts have been made to refine assessment and extend them to new populations. The Nature Connection Index (NCI; Richardson et al., 2019), the CN-12 scale (Hatty et al., 2020), the AIMES scale (Meis-Harris et al., 2021), and the Affective, Behavioural, and Cognitive Connection to Nature Scale (ABC-CNS; Cuadrado et al., 2023) represent attempts to update or expand measurement in line with contemporary needs. These newer scales illustrate the continuing effort to design instruments that are sensitive to different age groups, cultural contexts, and dimensions of human-nature relationships.

Identifying the similarities between the various elements about connection to nature facilitates more comprehensive and unified understanding, which allows consideration of nature connectedness as an overarching construct (Tam, 2013). Central psychological dimensions consistently emphasized across models include emotions, values, identity, and spirituality (Mayer and Frantz, 2004; Nisbet et al., 2009; Schultz, 2002), alongside behaviors, direct experiences, and time spent in nature (Clayton, 2003; Mayer et al., 2009). A defining feature of connectedness to nature is the perception of interdependence with the nonhuman world (Mayer and Frantz, 2004), which distinguishes it from environmentalism by encompassing elements of personal identity and lived interactions with the natural environments (Kovi et al., 2023). Within this broader perspective, Clayton (2003) notion of environmental identity remains foundational, conceptualizing identity with nature as a distinct component of self-concept.

Despite the variety of measures available, several issues and limitations associated with these instruments exist. To consolidate these issues and limitations and support a clearer comparison across instruments, Table 1 provides a comparative overview of key scales used to assess human-nature relationships. One major problem is the lack of a unified definition and conceptualization of nature connectedness, which leads to inconsistencies in the dimensions and factors assessed by different measures (Tam, 2013; Restall and Conrad, 2015). Because comparing the outcomes of differing research remains difficult, the lack of agreement has hindered the development of a comprehensive understanding of the human-nature relationship. Additionally, many of the measures used have been developed and validated mostly in Western, educated, industrialized, rich, and democratic (WEIRD) societies, which raises concerns about their cross-cultural applicability and generalizability (Clayton et al., 2021). However, even within WEIRD countries, there can be significant cultural, social, and environmental differences that may influence individuals’ connections to nature. For example, Australia’s unique landscapes, biodiversity, and Indigenous heritage may shape people’s experiences and relationships with nature in ways that differ from other WEIRD countries. Furthermore, some measures might not adequately capture the dynamic, context-dependent nature of individuals’ connections to nature, as they often rely on self-reported, trait-like assessments (Fretwell and Greig, 2019). Given the inconsistencies, standard measures of nature connectedness may need adjustment (Friedman et al., 2024).

**Table 1:** Comparative overview of scales assessing human-nature relationships.

Scale—author, year	Core conceptual focus	Primary dimension(s)	Notes/limitations identified in the literature
Emotional Affinity toward Nature (EATN)—Kals et al., 1999	Emotional tendency toward nature	Affective	Focuses on emotional affinity
Inclusion of Nature in Self (INS)—Schultz, 2001	Nature as part of self-concept	Identity Cognitive	Single item; Conceptualizes connection as self-nature overlap
Environmental Identity (EID)—Clayton, 2003	Nature integrated into identity	Identity Cognitive	Positions nature connectedness within self-concept
Connectedness to Nature Scale (CNS)—Mayer and Frantz, 2004	Emotional bond with nature	Intended affective	Items reflect cognitive appraisals rather than emotional experiences
Love and Care for Nature (LCN)—Perkins, 2010	Emotional attachment and care	Affective	Developed to address affective limitations of earlier measures
Connection to Nature Index (CNI)—Cheng and Monroe, 2012	Emotional affinity (youth-focused)	Affective	Explicit focus on affective connection
Dispositional Empathy with Nature (DEN)—Tam, 2013	Empathy toward nature	Affective/Empathic	Highlights empathy as a distinct dimension
Connectivity with Nature (CWN)—Dutcher et al., 2007	Relationship with nature	Affective Experiential	Captures affective and experiential connections
Nature Relatedness (NR)—Nisbet et al., 2009	Psychological relationship with nature	Self-perspective Experience	Includes cognitive aspects alongside affective
Commitment to Nature (COM)—Davis et al., 2009	Willingness to act for nature	Behavioral	Focuses on behavioral commitment
New Ecological Paradigm (NEP)—Dunlap et al., 2000	Environmental worldview	Attitudinal Cognitive	Measures attitude rather than connection
Disposition to Connect with Nature (DCN)—Brügger et al., 2011	General orientation toward nature	Dispositional	Captures broad orientation toward the natural world
Nature Connection Index (NCI)—Richardson et al., 2019	General nature connection	Affective Behavioral	Developed to refine and update assessment
CN-12—Hatty et al., 2020	Brief nature connection	Identity Experience Philosophy	Shortened measure extending prior scales
AIMES—Meis-Harris et al., 2021	Multidimensional experience	Affective Identity Meaning	Designed to extend assessment to new contexts
ABC-CNS—Cuadrado et al., 2023	Connection as ABC model	Affective Behavioral Cognitive	Explicitly integrates ABC dimensions

While these models and measures have substantially advanced understanding of human connection to nature, it is also important to acknowledge that the concept of connection to nature has not always been benign. Beyond its psychological an educational application, discourses of nature have at times been appropriated in ways that reinforced exclusionary practices and social hierarchies. Recognizing these legacies provides a more balanced perspective on the concept and prepares the ground for considering its problematic historical and contemporary issues.

### 1.4 Critical perspectives on connection to nature

While connection to nature has many proven benefits, within a historical context some researchers suggest it has been appropriated in ways that yielded harmful consequences (Merchant, 2003). This alleged misappropriation has been facilitated by a romanticized view of nature that ignores historical realities. While throughout time events such as extreme weather and disease have existed, humans have sought to develop technologies to escape such perils. The ability to control external environments and create shelters or control fire has parallels to today with the building of houses and controlling internal environments with air conditioning or heating.

The advancement of technology has increasingly insulated societies from direct natural threats. This distancing from natural threats evokes the concept of wilderness as “untrammeled by man” which can be considered to have been created to serve exclusionary purposes (Boerigter et al., 2025). The forced removal and exclusion of the original indigenous stewards during the founding of early national parks such as Yellowstone National Park in 1872 is evidence that the impact of creating a wilderness preserve caused alienation rather than connection to nature for some people (Kantor, 2007). Founding of national parks initially was primarily for non-indigenous tourism (Merchant, 2003). Evidence of this action is present in other nations throughout the same period. The establishment of Australia’s first national park, Royal National Park, in 1879 also occurred in the context of British colonialism and the dispossession of Aboriginal peoples from their traditional lands (Robin, 2013). The notion of “wilderness” as a pristine, untouched landscape has been increasingly questioned, as it overlooks the long history of indigenous presence and cultural connections to these lands (Kaye, 2021).

Despite these highly problematic historical and contemporary associations between love of nature and racial exclusion, nurturing a meaningful societal connection to nature is more critical than ever in today’s global context of a crises of climate change, mass extinction, and biodiversity loss. The challenge of dealing with these problematic associations lies in fostering connection to nature while remaining vigilant against its potential exploitation for exclusionary purposes. This requires acknowledging that connection to nature must be inclusive, recognizing the deep ecological knowledge and practices of indigenous communities who have been systematically excluded from ‘wilderness’ areas. Moving forward, environmental movements, today, must actively work to decolonize conservation practices rather than divide communities. While connection to nature is not an unproblematic concept, the advantages of such a connection outlined above merit strong consideration, provided it is pursued through approaches that promote equity, justice, and genuine inclusivity.

Yet unresolved questions remain especially in relation to the terminology and measurement of connection to nature. A wide range of terms such as “connection to nature”, “connectedness to nature”, “nature relatedness” and “inclusion of nature in self” have proliferated in the literature, all grounded in the Biophilia Hypothesis which indicates humans are evolutionarily pre-determined to be attracted to and feel connected with the natural world (Wilson, 1984). Although often used interchangeably, these terms emphasize overlapping but slightly different aspects of human affinity toward the natural environment. A further challenge lies in measurement of nature connectedness. Considering the limitations and contradictions across existing measures, it is plausible that the commonly used instruments may not adequately capture the experiences of younger generations. Friedman et al. (2024, p. 11) argue that “some of the commonly used measures of connection to nature might require adjustments to account for the nuance reflected in younger generations’ experiences”. This issue is particularly significant in adolescence in which connection to nature may differ from childhood in both form and trajectory. While existing models offer important conceptual contributions, variation in how nature connectedness is conceptualized and operationalized can limit the consistent capture of these developmental nuances. Parameters for measurements therefore need to be established in a conceptual framework focused on adolescent connection to nature that integrates existing models, consolidates overlapping dimensions, and reduces conceptual ambiguity. Such a framework would provide a foundation for developing instruments that more sensitively and accurately determine adolescents’ nature connectedness, enabling schools to evaluate the effectiveness of their environment-oriented programs in terms of facilitation of connection to nature. To support the development of a systematic framework the paper undertakes an integrative review and addresses the following research question: What are the key dimensions for inclusion in a framework for understanding and assessing adolescents’ nature connectedness?

## 2 Methodology

The present study employed an integrative literature review methodology to develop Integrated Nature Connectedness Framework (INCF), a comprehensive conceptual framework for understanding the dimensions of nature connectedness. An integrative literature review is a comprehensive approach that allows for the synthesis of literature from various disciplines and methodologies, enabling a more thorough understanding of complex phenomena (Whittemore and Knafl, 2005). It is also a valuable tool for advancing knowledge and driving research forward in the field (Elsbach and Van Knippenberg, 2020).

This methodology is particularly well-suited for the development of a new conceptual framework for nature connectedness, as it facilitates the integration of empirical literature from diverse fields such as education, psychology, and environmental studies. By combining findings from different research designs and methods, an integrative review can identify key concepts, theories, and gaps in the body of current literature, providing a solid foundation for the creation of a more inclusive and nuanced framework (Torraco, 2005). It can identify important themes, patterns, and relationships that individual studies may miss. The fresh perspectives offered by integrative reviews arise from the synthesis of the literature itself, rather than being guided by the authors’ preconceived ideas (Elsbach and Van Knippenberg, 2020). In this integrative review the literature was intentionally synthesized as a fit-for-purpose conceptual model of adolescent connection to nature by consolidating overlapping dimensions to reduce redundancies.

### 2.1 Search process

For the review, the literature search was conducted in August 2024 through the university library search engine, which provided access to multiple academic databases specified in the subsequent section. This comprehensive approach was crucial for capturing the interdisciplinary nature of research on nature connectedness, spanning education, environmental psychology, conservation science, health sciences, and other fields. The use of multiple databases ensured that our review scrutinized the diverse ways in which nature connectedness has been conceptualized and studied across different academic fields. The search strategy employed a combination of keywords aimed at capturing the various terminologies found in the literature regarding the concept of nature connectedness. We used the following search terms:

*(“connection to nature” OR “connection with nature” OR “connected to nature” OR “connecting to nature” OR “connected with nature” OR “connecting with nature” OR “nature connection” OR “connectedness to nature” OR “connectedness with nature” OR “nature connectedness” OR “nature connected” OR “nature relatedness”) AND (Quantitative OR Qualitative OR “mixed method”).*

### 2.2 Inclusion/exclusion process

Inclusion criteria

- peer-review journal articles and book chapters
- conceptual framework for nature connectedness
- empirical studies
- published from 2014 to 2024
- in English

The criterion of empirical studies meant that the conceptual framework had been operationalized and enabled assessment of the explanatory power of different conceptual frameworks. The investigative process involved carefully mapping the dimensions of nature connectedness as defined in each study’s conceptual framework, which were then systematically extracted and cataloged. Overlapping dimensions which are expressed through different terminology were subjected to comparative analysis and subsequently grouped under broader thematic categories. In cases where conceptually similar dimensions were articulated through different terminological designations, interpretive analytical procedures were implemented to identify underlying epistemological commonalities. This process enabled a more coherent synthesis of overlapping dimensions, allowing for greater conceptual clarity across otherwise fragmented terminologies.

To conduct a comprehensive and rigorous integrative literature review, this study allowed an iterative process involving concept mapping, peer review feedback, and cycles of improvement. Concept mapping was utilized to identify, categorize, and connect the main constructs associated with, and the dimensions of, nature connectedness. Peer review feedback among authors is an essential aspect of the research process that enhances the quality and credibility of the work (Horbach and Halffman, 2018). Feedback was incorporated at multiple stages of the review to provide insights, critique, and suggestions for improvement, ensuring the rigor and relevance of the emerging framework. The iterative cycles of improvement, moving from concept mapping to peer review feedback and back to concept mapping, constituted a third critical aspect of the methodology, ensuring that the framework evolved through a rigorous process of synthesis and validation.

### 2.3 Overview of included studies

**Figure 1:**
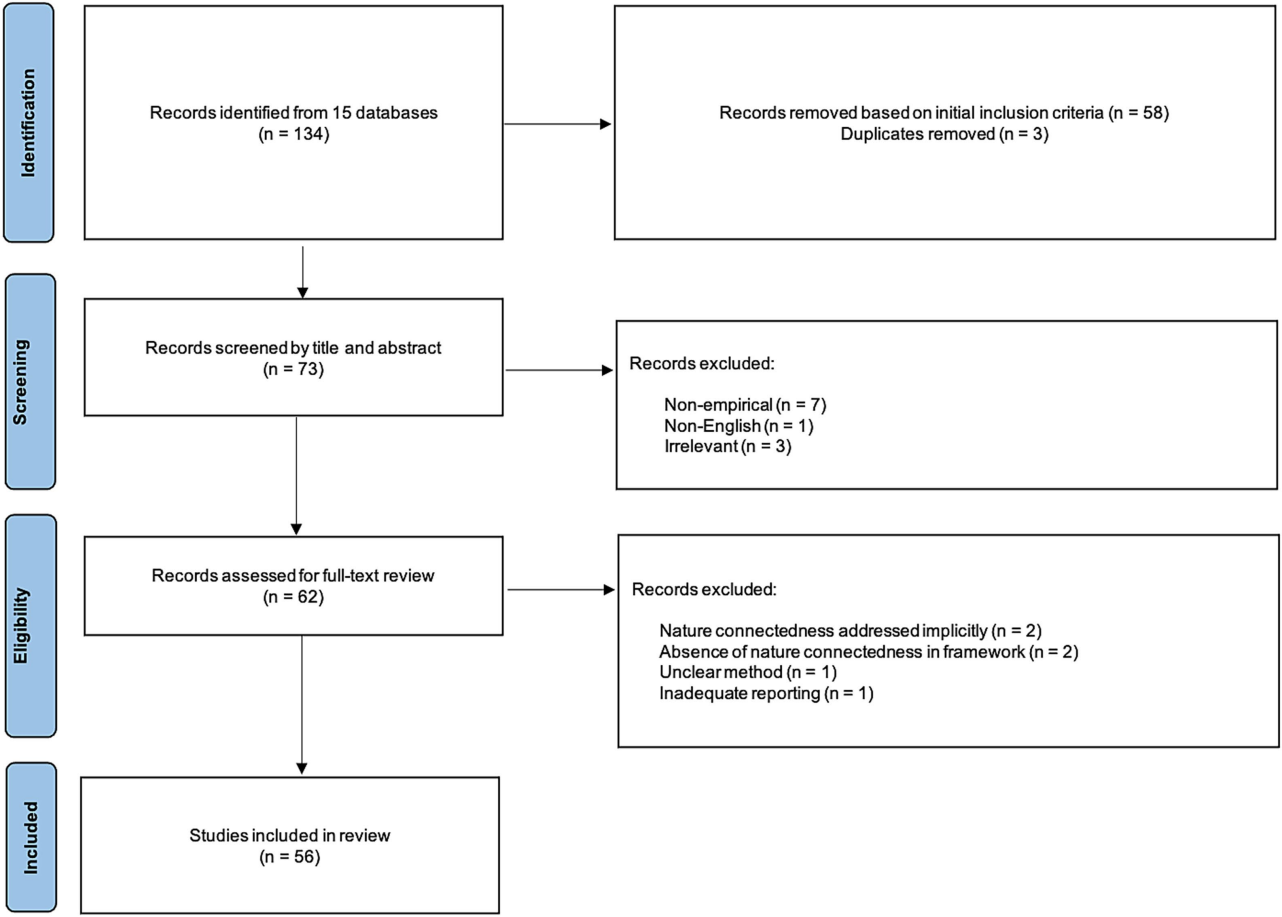
PRISMA flow diagram.

The search, screening, and reporting procedures of this integrative review approach were guided by PRISMA (Page et al., 2021) to enhance transparency and reproducibility. During the identification stage, a comprehensive search was conducted using the university’s library search engine with the keywords, resulting in a total of 134 studies, which were displayed across 15 databases that are Directory of Open Access Journals (DOAJ), EBSCOhost E-Journals, Elektronische Zeitschriftenbibliothek (EZB)–Frei zugängliche E-Journals, Gale Academic OneFile, GFMER Free Medical Journals, IngentaConnect Journals, ProQuest Central, Publicly Available Content Database, PubMed, ROAD (Directory of Open Access Scholarly Resources), Science Citation Index Expanded (Web of Science), Scopus, Social Sciences Citation Index (Web of Science), Taylor and Francis Online, and Unpaywall. Following the removal of duplicates and the application of initial inclusion criteria, 73 studies were retained for screening. In the screening stage, these studies were uploaded to a software program called Covidence so that the authors could conduct a blind screening of the studies based on the abstract and title. Subsequent to five instances of conflict between authors, the two studies were selected for the review and three studies were excluded. During the eligibility stage, 62 studies were deemed to meet the inclusion criteria and were subjected to a full-text review. Then, six additional studies were excluded for methodological concerns or unclear conceptual frameworks, resulting in 56 final studies for further analysis (see Figure 1).

## 3 Results

Of the 56 studies, 27 employed a mixed-method approach, 16 adopted quantitative approaches only, and 13 used qualitative methods only. These methodological modalities reflect the different ways researchers have sought to understand nature connectedness, whether through surveys, interviews, observations, or combination of these methods. Psychometric scales featured most prominently (*n* = 51), followed by the interviews (*n* = 22) and survey items (*n* = 8). Additionally, smaller number of reviewed studies utilized observations (*n* = 4), focus groups (*n* = 2), cognitive mapping (*n* = 1), field diaries (*n* = 1), and even informal conversations via SMS (*n* = 1), showing both conventional and innovative approaches to data gathering. Among the psychometric scales, 16 distinct types were employed. Of the 40 studies that used scales to measure nature connectedness, 33 incorporated one scale, five used two scales, and two employed four scales in their measurement protocols, with a total of 51 scales used. Certain scales were prevalent, most notably Nature Relatedness (NR) Scale (*n* = 10; Nisbet et al., 2009), Inclusion of Nature in Self (INS) Scale (*n* = 8; Schultz, 2002), Nature Relatedness-6 (NR6) Scale (*n* = 7; Nisbet and Zelenski, 2013), and Connectedness to Nature Scale (CNS; *n* = 5; Mayer and Frantz, 2004), yet a wider range of infrequently utilized instruments was also documented in Table 2.

**Table 2:** Frequency of NC-specific data collection methods used in the reviewed studies.

NC-specific data collection method	Frequency
Data collection method	
Scales	51
NR Scale (Nisbet et al., 2009)	10
Inclusion of Nature in Self Scale (INS; Schultz, 2002)	8
NR-6 Scale (Nisbet and Zelenski, 2013)	7
Connectedness to Nature Scale (CNS; Mayer and Frantz, 2004)	5
7-item Version of the Connectedness to Nature Scale (CNS; Pasca et al., 2017, as cited in, Garza-Teran et al., 2022)	2
Environmental Identity Scale (EID; Clayton, 2003)	2
State Connectedness to Nature Scale (Mayer and Frantz, 2004; Mayer et al., 2009)	2
Nature Connection Index (Richardson et al., 2019)	2
Love and Care for Nature Scale (Perkins, 2010)	1
Connection to Nature Index for Parents of Preschool Children (CNI-PPC; Sobko et al., 2018, as cited in, Barrable and Booth, 2020)	1
Love and Care for Nature–Rural (LCNR) Scale (Mikolajczak et al., 2019)	1
Connectedness to Nature Scale (Olivos et al., 2011, as cited in, Schueler and Newberry, 2020)	1
Children’s Environmental Perceptions Scale (CEPS; Larson et al., 2011, as cited in, Williams et al., 2021)	1
AIMES Scale (Meis-Harris et al., 2021)	1
HaN Scale—Sense of Human–Nature Relationships (Van den Born, 2008, as cited in van Heel et al., 2024)	1
Other rating scales (context-specific items)	6
Interview	22
Survey items (open-ended questions, categorical response items)	8
Observation	4
Focus group	2
Cognitive mapping	1
Field diaries	1
Informal conversation via SMS	1

31 studies used one of these above instruments and 25 studies used two or more of above data collection method. Single-instrument studies followed either purely quantitative (Likert scale) or purely qualitative (interview) approach. Other studies either combine different types of quantitative scales, integrated quantitative and qualitative approaches (e.g., scales with interviews), or employed comprehensive measurement strategies using multiple instruments across both quantitative and qualitative domains.

In parallel with the variation in research design and data collection techniques, participant demographics also exhibited notable variation in terms of age, spanning a wide age spectrum ranging from 1 to 90 years of age. Nonetheless, despite the broad age represented, fewer than a quarter (21%) of the studies centered on individuals only under 18. The vast majority of these youth-oriented studies were published between 2019 and 2024, indicating a relatively recent scholar shift toward the younger populations. This demographic pattern is reflected in the ‘conceptual foundations’ in Table 3, where four of the frameworks were specifically designed for use with children and adolescents, while 12 were intended for both adolescents and adults, and the remaining frameworks were originally developed for use with adult populations. The recent increase in youth-focused studies suggest growing recognition of the need for developmentally appropriate research approaches, though the conceptual foundations may still be catching up to this empirical shift.

**Table 3:** Analysis and synthesis of dimensions of NC.

Published dimensions	Conceptual foundation (CF)	Number of studies using CF	Consolidated dimension	Sub-dimension	Definition
NR-self > feelings	Nisbet et al., 2009 *(Adolescents and adults)*	10	ERC-Emotional and Relational Connection	EMO-Emotional Attachment	Both positive and negative feelings toward nature.
Emotional	Ives et al., 2018 *(Not age-specific)*	7			
Heart	Interviews and observations *(Adults)*	1			
Affective > love, joy	Emotional nature connection (LCNR-rural) Mikolajczak et al., 2019 *(Adults)*	1			
Enjoyment	Cheng and Monroe, 2012 *(Children)*	3			
Empathy	Cheng and Monroe, 2012 *(Children)*	3			
Emotional attachment	Based on adolescents’ experience—interviews	1		INT-Integration with Nature	Creating a sense of harmony and unity with nature.
Sense of affiliation	Boiral et al., 2019 *(Not age-specific)*	1			
Compassion	Added after interviews to Ives et al.’s (2018) framework *(Adults)*	1			
Emotional	Interview *(Adults)*	1			
Integration with nature	Mayer and Frantz, 2004 *(Adolescents and adults)*	2			
Sense of belonging	Schultz et al.’s (2004, as cited in Garza-Teran et al., 2022) definition “the sense and level of belonging humans have with the natural world”	1			
Identity > passion	Interview *(Adults)*	1			
NR-self > thoughts	Nisbet et al., 2009 *(Adolescents and adults)*	10	CC-Cognitive Connection	COG-Cognitive Attributes	Knowledge and awareness of environmental systems, coupled with the ability to think critically and analytically about ecological issues and relationships.
Self	Olivos et al.’s (2011, as cited in Schueler and Newberry, 2020) definition “The relationship between one’s self-image and the natural world” *(Not age-specific)*	1			
Sense of oneness	Mayer and Frantz’s (2004) definition *(Not age-specific)*	2			
Identity > environmental perspective	Interview *(Adults)*	1			
Part of nature	Mayer and Frantz, 2004 *(Adolescents and adults)*	4			
Sense of oneness	Cheng and Monroe, 2012 *(Children)*	2			
Cognitive	Ives et al., 2018 *(Not age-specific)*	8			
Cognitive	Schultz’s (2002) definition *(Not age-specific)*	3			
Cognitive	Garza-Teran et al., 2022 *(Not age-specific)*	1			
Mind	Interview and observation *(Adults)*	1			
Awareness	Cheng and Monroe’s (2012) *(Children)*	1		KNW-Knowledge of natural Places	Familiarity with local environments and natural resources.
Knowledge of natural places	Based on the wellbeing indicators by Sarriera et al., (2014, as cited in Galli et al., 2017) *(Children)*	1			
Symbolic meaning-making > self-achievement	Based on adolescents’ experience—interview	1			
Innate or evolutionary	Interview *(Adults)*	1			
Experiential	Ives et al., 2018 *(Not age-specific)*	5	ESE-Experiential and Sensory Engagement	SOM-Somatic Nature Connection	The instinctive bond with nature through both conscious sensory engagement and unconscious bodily sensations and intuitive responses.
NR-experience	Nisbet et al., 2009 *(Adolescents and adults)*	10			
Experience of nature	Added by Sedawi et al. (2020) to Cheng and Monroe’s (2012) *(Children)*	1			
Material	Ives et al., 2018 *(Not age-specific)*	5			
Frequency of visits/ contact with nature	Based on the wellbeing indicators by Sarriera et al., (2014, as cited in Galli et al., 2017) *(Children)*	1			
Sensory engagement	Based on adolescents’ experience—interview	1			
Body	interview and observation *(Adults)*	1		MARE-Material-Recreational Engagement	Active involvement with the natural world through outdoor leisure activities and the direct use or consumption of natural resources.
Behavior > time spent outdoor	Interview *(Adults)*	1			
Outside regardless of whether	Dimensions of nature connection (Grimwood et al., 2018) *(Adults)*	1			
Behavior > recreation	Interview *(Adults)*	1			
Philosophical	Ives et al., 2018 *(Not age-specific)*	5	PES-Philosophical-Ethical-Spiritual	PHET-Philosophical and Ethical Perspectives	The foundational worldviews and moral philosophies that shape our understanding of humanity’s relationship with nature, and our ethical responsibilities.
NR-perspective	Nisbet et al., 2009 *(Adolescents and adults)*	8			
Attitude toward nature	Dimensions of nature connection (Grimwood et al., 2018) *(Adults)*	1			
Attitudes toward preserving nature	Based on the wellbeing indicators by Sarriera et al., (2014, as cited in Galli et al., 2017) *(Children)*	1			
Symbolic meaning-making > esthetic experience	Based on adolescents’ experience—interviews	1			
Spirit	Interview and observation *(Adults)*	1			
Identity > spirituality	Interview *(Adults)*	1		SPR-Spirituality and Symbolic Appreciation	A deep, personal bond with nature combined with recognition of its esthetic and intrinsic value, involving both spiritual/transcendent experiences in natural settings and appreciation of nature’s inherent worth.
Ethics	Interview *(Adults)*	1			
Affective > beauty	Emotional nature connection—(LCNR-rural) Mikolajczak et al., 2019 *(Adults)*	1			
Behaviors to help preserve	Based on the wellbeing indicators by Sarriera et al., (2014, as cited in Galli et al., 2017) *(Children)*	1	SPS-Sustainable Practices and Stewardship	SUS-Sustainable Practices	The practical implementation of environmental responsibility through specific behaviors and lifestyle choices.
Sense of responsibility (and concern)	Cheng and Monroe, 2012 *(Children)*	3			
Stewardship	Dimensions of nature connection (Grimwood et al., 2018) *(Adults)*	1			
Behavior > sustainable behaviors	Interview *(Adults)*	1			
Willingness to act	Interview *(Adults)*	1			
Commitment	Added after interviews, to Ives et al.'s (2018) framework *(Adults)*	1		STW-Stewardship Commitment	The sense of duty and active commitment to protect, respect, and care for natural environments and local green spaces.
Care	Added after interviews, to Ives et al. (2018) framework *(Adults)*	1			
Symbolic meaning-making > psychological restoration	Based on adolescents’ experience —interview	1	PPW-Psychological and Physical Wellbeing	PSY-Psychological Wellbeing	Psychological wellbeing experiences from nature interaction.
Affective > psychological wellbeing	Emotional nature connection—(LCNR-rural) Mikolajczak et al., 2019 *(Adults)*	1			
Resilience	Dimensions of nature connection (Grimwood et al., 2018) *(Adults)*	1		PHY-Physical Wellbeing	Embodied experiences of wellbeing along with psychological wellbeing.

Across the 56 reviewed studies, 30 studies conceptualized NC as a single construct that was multidimensional in nature, while the remaining studies treated it as unidimensional. We analyzed the studies adopting a multidimensional approach, identified the constituent dimensions of NC, and examined the underlying conceptual foundations together with the number of studies drawing upon each foundation. As presented in Table 3, the analysis revealed 43 distinct dimensions across these studies. This dimensional landscape was subsequently synthesized into a refined framework encompassing six core dimensions and 12 subdimensions.

Among the dimensions examined, two identified by Grimwood et al. (2018) could not be incorporated into the framework. These were “access to/use of nearby nature” described as opportunities to explore urban nature with newly developed awareness, skills, and habits, and “learning and facilitation style” articulated through practices such as questioning, playing, observing, and core routines. This exclusion was deemed necessary because they primarily reflect external environmental conditions and pedagogical strategies rather than intrinsic aspects of nature connectedness. However, other dimensions from the same study, namely *time in nature, attitude toward nature, resilience,* and *stewardship* were incorporated (see Table 3). In addition, unidimensional studies were included if they either specified particular dimensions or provided clear definitions of connection to nature (e.g., Kim et al., 2023; Rosa et al., 2022). By contrast, studies that either lacked explicit definitions or did not indicate the particular dimension of NC examined were excluded from Table 3, as their conceptual boundaries remained unclear and could not be meaningfully integrated into our framework (e.g., Yerbury and Weiler, 2020; Michaelson et al., 2020; Fehnker et al., 2022).

### 3.1 Constructs associated with nature connectedness

The empirical literature demonstrates robust associations between nature connectedness and a spectrum of other constructs. Showing these links provides insight into the applied significance of nature connectedness and the diverse domains in which it has been positioned. The construct analysis focused on the ones that appeared in five or more studies, as these were deemed to offer sufficient conceptual weight to shape the themes. Most of these constructs fall under the themes such as *personal wellbeing, environmentally friendly behaviors, human-nature relationships, educational practices, environmental sensitivity and perception, self-concept and identity*, and *personal values and traits.* To ensure conceptual rigor, a frequency threshold criterion (*n* ≥ 5) was judiciously applied in accordance with established thematic analysis protocols as outlined in Braun and Clarke (2021) study. Constructs with lower frequencies (*n* < 5) were not included in the main text to preserving the analytical focus on the most substantiated thematic relationships, and these constructs are located in the Supplementary materials.

*Wellbeing* emerged as the most frequently associated construct (*n* = 18), with substantial evidence linking nature connectedness to positive psychological states including enhanced mental health (Bass and Loeffler, 2023) and reduced anxiety (Martyn and Brymer, 2016). The second-most frequent theme, *environmentally friendly behaviors* (*n* = 15), also reveals numerous connections, with pro-environmental behaviors representing the strongest association, complemented by more specific behavioral manifestations such as pro-conservation behaviors (Butler et al., 2024), green purchase intentions (Aruta and Pakingan, 2022), environmental stewardship (Bass and Loeffler, 2023), and sustainable dietary choices (Stott et al., 2024). The next most prominent theme evidences significant associations between nature connectedness and various forms of *human-nature interactions* (*n* = 14), encompassing urban nature experiences (Church, 2018), contact with nature (Garza-Teran et al., 2022), and more specialized engagements such as marine wildlife experiences (Yerbury and Weiler, 2020), and nature-based recreation (Rosa et al., 2022). *Educational practices* (*n* = 12) also show substantial linkages, including environmental citizen science (Williams et al., 2021), urban outdoor educational practices (Grimwood et al., 2018), nature nursery (Barrable and Booth, 2020), forest school training (Cont et al., 2023), and wilderness expedition (Barton et al., 2016). *Environmental sensitivity and perception* (*n* = 8) represents another significant theme, incorporating constructs such as eco-centric perspective (Yerbury and Weiler, 2020), perception of nature (Duvernoy and Gambino, 2022), environmental consciousness (Yurtsever and Angin, 2022), and soundscape perception (Francomano et al., 2022). *Self-concept and identity* (*n* = 5) and *personal values and traits* (*n* = 5) themes include the studies that reveal the deeper psychological underpinnings of nature connectedness with the constructs of self-esteem (Barton et al., 2016), self-identity (Kim et al., 2023), empathy (Schueler and Newberry, 2020), personal values (Mikolajczak et al., 2023), and characteristic strength (Rokhmah et al., 2024). More specialized but nonetheless significant associations emerge with the themes including knowledge (*n* = 4), technology (*n* = 4), mindfulness practices (*n* = 3), environmental management (*n* = 3), external factors (*n* = 3), social responsibility (*n* = 2), altruism (*n* = 2), appropriation of space (*n* = 2), and unique constructs such as city design (Church, 2018), connectedness to animals (Setti et al., 2022), physical activity (Teixeira et al., 2023), childhood experiences (Fretwell and Greig, 2019), and strategic thinking (Karami and Gorzynski, 2022).

Collectively, these associated constructs point to the considerable applied significance of nature connectedness. Rather than being confined to an internalized disposition, nature connectedness has been examined in relation to diverse domains as seen in previous paragraphs. To situate these associated constructs more clearly, it is necessary to turn to the ways in which the concept of nature connectedness itself has been conceptualized, beginning with its terminological variation and the definitional scope, and dimensions through which it has been operationalized.

This comprehensive review has illuminated the terminological diversity and definitional variation in relation to NC. Analysis of dimensional approaches, informed by both unidimensional (*n* = 26) and multidimensional (*n* = 30) studies, revealed considerable variation in the structuring and conceptual grounding of multidimensional conceptualizations. Building upon this analysis, the published dimensions were synthesized into Integrative Nature Connectedness Framework (INCF), comprising six consolidated dimensions, each with two subdimensions.

### 3.2 Terminology and definitions

The literature revealed a considerable diversity in articulating the human-nature relationship. “Nature connectedness” appears most frequently (*n* = 13), serving as a comprehensive construct encompassing the emotional, cognitive, and physical dimensions of how humans relate to natural environments (Grimwood et al., 2018). It explores how individuals incorporate natural elements into their self-concept (Garza-Teran et al., 2022) and represents a sophisticated psychological phenomenon, with particular emphasis on how people develop and maintain relationships with nature. Similarly, albeit with subtly divergent emphasis, “connectedness to nature” (*n* = 9) pertains to the affective bond between individuals and the natural world (Mayer and Frantz, 2004; Rosa et al., 2022) and examines the sense of ecological community and belonging (Furness, 2021). Meanwhile, “nature relatedness” (*n* = 10) refers to one’s appreciation and understanding of our interconnectedness with all other living things (Nisbet et al., 2009; Lankenau, 2018). In addition, “connection to nature” (*n* = 9) refers to a subjective sense of physical, cognitive, emotional, or spiritual connectedness with the natural world (Karami and Gorzynski, 2022) and similar to “connectivity with nature”, a term that appeared in one study only (Surzykiewicz et al., 2023). Researchers characterize “connection to nature” as a quantifiable, trait-like construct exhibiting temporal and situational stability, though not immutable (Fretwell and Greig, 2019; Nisbet et al., 2009). Beyond these predominant terms, the literature includes “nature connection” (*n* = 5), “connection with nature” (*n* = 5), “connectedness with nature” (*n* = 2), and “affective relationships with nature” (*n* = 1) which specifically delineating individuals’ emotional inclination to nature. Moreover, the “human-nature connectedness” (*n* = 1) and “human-nature connection” (*n* = 1) were also used to better understand the relationship between humans and nature.

In effect, the academic corpus characterizes these diverse terminologies as “a patchwork of entangled terms” (Grimwood et al., 2018, p. 206), reflecting the inherent complexity of the human-nature relationship that resists reduction to singular or uniform definitions. In many instances, scholars appear to be describing essentially similar phenomena under different labels, slightly different emphases, or subtly varied operationalizations. While the effort to delineate between the terms is commendable, a closer look reveals considerable conceptual overlap. Yet without greater rigor in definitions, there is a risk that the field will continue to reproduce conceptual vagueness, thereby limiting its theoretical and practical impact.

Owing to the conceptual entanglements and nuanced overlaps apparent across the reviewed terms, this paper will adopt the most frequently used term, *nature connectedness (NC),* as its principal articulation, reflecting the multifaceted character of individuals’ relationships with nature.

### 3.3 Dimensions

Of the studies that used the term, almost half (*n* = 26) employed NC explicitly as a unidimensional construct, while acknowledging its recognized complexity. Numerous studies with a unidimensional concept used established metrics without questioning dimensionality and treated the construct as a cohesive psychological entity (e.g., Spangenberger et al., 2022; Yurtsever and Angin, 2022). Some researchers explicitly articulate their approach to NC by using Schultz (2002) definition as “the extent to which an individual includes nature within their cognitive representation of self” (Barton et al., 2016; Dussler and Deringer, 2020; Bezeljak et al., 2023), or Mayer and Frantz (2004, p. 504) definition as “experiential sense of oneness with the natural world” (Teixeira et al., 2023; Kim et al., 2023), or “the extent to which people believe themselves to be part of the nature” (Mikolajczak et al., 2023), emphasizing a cognitive understanding of self-nature integration. Others frame nature connectedness primarily through affective dimensions, conceptualizing it as capturing the feelings associated with experiences with nature (Barrable et al., 2024). These definitions might appear to be conceptualizing different constructs; however, research show a substantial convergence between these ostensibly distinct conceptualizations, suggesting they indeed assess a common underlying construct (Tam, 2013). For instance, some studies confirm that the 7-item (CNS-7) and 13-item (CNS-13) versions of the Connectedness to Nature Scale (Rosa et al., 2022), and the short-form Nature Relatedness scale (NR-6; Aruta and Pakingan, 2022) demonstrate robust unidimensional structures through confirmatory factor analysis. The prevalence of unidimensional frameworks likely stems more from pragmatic measurement considerations than robust theoretical justifications. A considerable portion of published empirical studies in this field tend to acknowledge complexity but operationalize simplicity through unidimensional framing. While unidimensional conceptualizations offer notable parsimony, they risk theoretical oversimplification through reductive approach to a manifestly complex psychological construct.

In contrast, this integrative review found that slightly more than half of the studies (*n* = 30) preferred frameworks for NC with multiple dimensions. Our analytical process revealed 43 distinct dimensions, describing various facets of nature connectedness (see Table 3). Within this conceptual abundance, some dimensions represented similar psychological phenomena articulated through different nomenclature. For instance, unified multi-dimensions included: *cognitive* (Helne, 2022), *awareness* (Fretwell and Greig, 2019), *knowledge of natural places* (Galli et al., 2017), and *mind* (Karami and Gorzynski, 2022); *emotional* (Francomano et al., 2022), *affective* (Mikolajczak et al., 2023)*, heart* (Karami and Gorzynski, 2022), *enjoyment* (Barrable and Booth, 2020), and *emotional attachment* (Tseng and Wang, 2020); *and NR-experience* (Cont et al., 2023), *frequency of visits* (Galli et al., 2017), *experience of nature* (Sedawi et al., 2020), and *experiential* (Surzykiewicz et al., 2023). To navigate this complexity, we employed an iterative process of conceptual synthesis, beginning with a comprehensive cataloging of each dimension precisely as articulated in its original literature. This was followed by meticulous cross-comparison, examining semantic proximities, conceptual intersections, and fundamental distinctions. As taxonomic structure evolved, certain overarching dimensions emerged.

This dimensional synthesis led us to develop an integrative framework, providing a comprehensive architecture for understanding nature connectedness that accommodates 43 dimensions identified in the literature. By reconciling seemingly disparate constructs into coherent multidimensional model, we offer conceptual clarity and methodological guidance.

## 4 Discussion

### 4.1 The integrated nature connectedness framework

Drawing upon the comprehensive analysis above, we propose an integrative framework that synthesizes the complex, multidimensional character of nature connectedness and is explicitly intended for adolescents. The Integrated Nature Connectedness Framework (INCF) emerged from meticulous examination and iterative refinement of 43 distinct dimensions identified in 56 studies. These were consolidated into six core dimensions, each comprising two distinct but complementary subdimensions (Table 4).

**Table 4:** Integrated nature connectedness framework.

Integrated nature connectedness framework		
Consolidated dimensions	Subdimensions	Definition
Emotional and relational connection	Emotional attachment	Positive and negative feelings toward nature
	Integration with nature	Sense of harmony and unity
Cognitive connection	Cognitive attributes	Understanding ecological systems and thinking critically
	Knowledge of natural places	Familiarity with local environments
Experiential and sensory engagement	Somatic nature connection	Bodily and sensory engagement with nature
	Material-recreational engagement	Active, hands-on interaction with nature through practical and restorative activities.
Philosophical-ethical-spiritual connection	Philosophical and ethical perspectives	Foundational beliefs about human-nature relationship
	Spiritual and symbolic appreciation	Personal bond and appreciation of nature
Sustainable practices and stewardship	Sustainable practices	Environmental responsibility through actions
	Stewardship commitment	Sense of duty and active commitment to protect natural spaces
Psychological and physical wellbeing	Psychological wellbeing	Psychological wellbeing experiences
	Physical wellbeing	Embodied experiences of wellbeing

#### 4.1.1 Emotional and relational connection

*Emotional and relational connection* (ERC) represents a meaningful bond with nature characterized by a spectrum of emotions and a sense of unity, reflecting both affective experiences and the perception of harmony with the natural world. The relational aspect of this dimension extends beyond mere emotional reactions and focuses on how individuals experience nature not as an inert object but as an active participant in a reciprocal relationship which implies embeddedness with the natural world or a process of integration into it. This dimension, evident in 33 studies as seen in Table 3, has two subdimensions*: emotional attachment* (EMO) and *integration with nature* (INT).

EMO is a significant aspect of the concept of an individual’s relationship with the natural world, encompassing a range of feelings about nature. Qualitative data highlights numerous feelings elicited by encounters with nature including being alive, feeling okay, relaxation, joy, happiness, contentment, love, appreciation, compassion, fascination, interest, sadness, disappointment, concern, anxiety, disgust, fear, and anger (Martyn and Brymer, 2016; Stott et al., 2024; Lankenau, 2018; Butler et al., 2024). While some studies suggest the negative emotions might potentially offset some positive effects of nature connectedness (Fretwell and Greig, 2019), others contend that any emotional experience can be associated with increased nature connectedness (Butler et al., 2024). Categorizing emotions as positive or negative is reductive, considering that emotions that are viewed as negative (such as disgust) might prompt individuals to take positive action.

The feeling that nature is not separate, but part of one’s identity or seeing the self as intertwined with nature is reflected by INT subdimension. Nature connectedness can be described as one’s feeling of “oneness” with nature that stems from accepting nature as an important part of one’s self-definition (Mayer and Frantz, 2004). This is distinguished from the cognitive connection by its emphasis on how people feel integrated with nature rather than what they believe about such integration. It is measured with the item “I often feel a sense of oneness with the natural world around me” in CNS (Mayer and Frantz, 2004), and “I feel like I am part of the natural world” in the revised CNI (Whitburn et al., 2023). “Part of who I am” is also closely align with indigenous worldviews characterized by their fundamental emphasis on holistic unity. Indigenous communities generally conceive of themselves and their natural environment as constituents of an extended ecological kinship network, a perspective that Salmon (2000) has termed “kincentric ecology” or in short “kincentricity.” While this conceptualization has a broad applicability, it has a particular significance within the Australian context, where kincentric ecology represents “a common thread” among Australia’s Indigenous peoples (Kearney et al., 2019). This paradigm transcends the Western dichotomy between human and non-human realms, and challenges anthropocentric frameworks, instead positioning people within interconnected web of reciprocal relationships.

ERC develops through the synthesis of cognitive understanding and experiential encounters, transforming factual knowledge and sensory data into personally meaningful relationships characterized by affective investment and relational identification with natural entities. For adolescence specifically, ERC assumes heightened developmental significance due to several interrelated processes characteristic to this life stage.

During adolescence, the intensified emotional experiences and heightened sensitivity to both social and environmental stimuli make this dimension particularly significant for emotional regulation and psychological wellbeing (Chawla, 2020). Importantly, the relational bonds may support emotional regulation not because nature serves instrumentally as a therapeutic tool, but because caring relationships themselves provide a source of emotional grounding (Chawla, 2020). Natural settings therefore may function as emotionally regulating environments where adolescents process intense feelings more constructively, with sensory experiences in nature helping to mitigate stress and anxiety (Martyn and Brymer, 2016; Barrette et al., 2025). Over time, recurrent experiences of positive feelings in natural environments may position nature as both personally significant and identity relevant. This integration might be conceptualized as the formation of an environmental identity (Clayton, 2003) or as the inclusion of nature in the self-concept (Schultz, 2002), whereby natural entities become psychologically affiliated with one’s self-representation. In this way, ERC may contribute to the longer-term consolidation of adolescents’ relational orientations toward the natural world.

#### 4.1.2 Cognitive connection

*Cognitive connection* (CC) addresses the intellectual and conceptual facets of how people understand their relationship with nature. This dimension, evident in 37 studies as seen in Table 4, has two subdimensions: *cognitive attributes* (COG) and *knowledge of natural places* (KNW). CC is articulated in two interrelated ways. First, as perceptions of people’s place in nature shaped by their underlying value orientations, ranging from egoistic to altruistic and biospheric (Mikolajczak et al., 2023). Second, as an aspect of self-concept, whereby individuals incorporate nature into their fundamental understanding of self, expressed in Schultz (2002, p. 67) description of “the extent to which an individual includes nature within his/her cognitive representation of self”. Together, these perspectives shape how people conceptualize environmental relationships and their place within ecological systems. In addition, CC encompasses environmental awareness, assessed through indicators such as whether children “notice birds and other sounds in nature” and “notice wildlife” (Barrable and Booth, 2020, p. 4); environmental knowledge to satisfy the mind’s curiosity and increase knowledge (Helne, 2022); and the capacity for critical reflection (Galli et al., 2017).

COG encompasses the developing knowledge structures, expanding awareness patterns that shape how adolescents perceive, categorize, and interpret natural phenomena. This dimension includes cognitive architecture through which environmental information is processed and understood, establishing the intellectual foundation for meaningful human-nature interaction during adolescence when abstract thinking capabilities are rapidly expanding. Improving abstract thinking and enhanced cognitive capacities enable young people to integrate nature into their self-concept and to construct complex mental models regarding environmental interdependence (Chawla, 2020). However, while engaging in all these efforts particularly in an era where children are increasingly growing up immersed in technology and surrounded by constant distractions, it is crucial that they do not succumb to cognitive fatigue. At this point, a growing body of research supports the Attention Restoration Theory, which posits that natural environments offer a form of ‘soft fascination’ that gently captures one’s attention, thereby facilitating mental rejuvenation (Kaplan, 1995). These studies suggest that exposure to natural environments promotes cognitive recovery and enhances attentional functioning (Moll et al., 2022).

Research also shows that as adolescents mature, their ability to comprehend ecological processes and human-nature interdependence improves, so cognitive growth aligns with their developing capacity for self-reflection and identity formation (Cheng and Monroe, 2012). The dynamic interplay between cognitive engagement and identity is critical, as it enables adolescents to see themselves as integral members of a broader ecological community, thereby fostering a sense of responsibility that transcends mere knowledge acquisition (Bowers et al., 2021). Building on this cognitive-identity linkage, adolescence is also characterized by increasing autonomy-seeking which is often reflected in decision-making processes and expressing individual viewpoints (Seiffge-Krenke and Pakalniskiene, 2011). In this developmental context, cognitive connection with nature constitutes a core dimension of nature connectedness as adolescents actively interpret ecological systems, evaluate human-nature interdependence, and reflect on personal values in relation to the natural world.

Here, we deliberately isolated KNW as a discrete subdimension of CC. This methodological decision represents a significant departure from existing literature, wherein it was not explicitly articulated as a distinct dimension, except one study which states that knowledge (and use) of neighborhood resources is associated with wellbeing in children and adolescents (see Galli et al., 2017). Our rationale is that localized ecological knowledge represents a unique facet of CC because when people possess specific knowledge about their local natural environments, they engage with nature through a different cognitive schema than those with merely abstract environmental knowledge. Knowledge of natural places in neighborhood functions as a crucial cognitive bridge, transforming distant appreciation into intimate connection because it is embedded in one’s lived experience, no longer abstract but rooted in a tangible relationship with their immediate environment.

#### 4.1.3 Experiential and sensory engagement

*Experiential and sensory engagement* (ESE) denotes a tangible relationship with nature developed through sensory awareness, bodily intuition, and active participation, emphasizing direct experiences and practical engagement with the natural world. In this framework, ESE is conceptualized as a constitutive dimension of nature connectedness. It captures how connection is embodied and enacted through experiences rather than resulting automatically from exposure alone. This dimension, evident in 27 studies as seen in Table 3, includes two subdimensions: *somatic nature connection* (SOM) and *material-recreational engagement* (MARE). While the former represents the instinctive bond with nature through both conscious sensory engagement and unconscious bodily sensations and intuitive processes, the latter captures an active involvement with the natural world through outdoor activities and direct use or consumption of natural resources.

SOM recognizes that human-nature relationships operate through visceral, corporeal experiences that engage the full spectrum of sensory modalities: touch, sound, smell, and sight. Connection to nature extends beyond the cognitive realm or *mind* to include *body*, and is described as an embodied personal connection (Karami and Gorzynski, 2022). The more various senses engaging, the deeper the connection with nature. The sense of touch, for example, is described not merely as a superficial interaction but as generating a profound “overall feeling of closeness to nature”, while unfamiliar or interesting sounds from nature are perceived as directing attention outwards and potentially cultivate a “feeling of oneness” with nature (Tseng and Wang, 2020, p. 124). The sense of smell, too, is noted for its capacity to “awaken attention and spirit” (Tseng and Wang, 2020, p. 124) and evoke potent memories, such as a smell of cut grass (Stott et al., 2024). Similarly, sight helps individuals appreciate the beauty of nature and can generate positive emotions (Tseng and Wang, 2020). Such connections manifest through immediate physical responses to natural or natural-like environments, where the body serves as both receptor and interpreter of ecological information. Direct physical contact or interactions with nature may foster the emotional and personal aspects of nature experiences (Fretwell and Greig, 2019; Duke and Holt, 2023). A pertinent contemporary example of this dimension can be the practice of forest bathing, characterized as a somatic engagement with forest environments that has garnered considerable scholarly and public attention (Chen et al., 2025). Empirical evidence demonstrates that immersive natural encounters effectively reduce anxiety, depression, and nature-deficit symptoms, while fostering profound tranquility through multisensory engagement. Unlike recreational engagements, it is distinguished by a conscious intention to foster healing connection with nature (Keller et al., 2024). Moreover, greater awareness of ongoing bodily processes (interoceptive awareness) enhances an individual’s ability to regulate emotions in response to negative feelings (Branham, 2024).

MARE represents active involvement with nature through outdoor activities and direct use or consumption of natural resources. This subdimension captures the tangible ways rooted in direct experiences with nature, with outdoor recreational activities highlighted as prominent examples such as hiking, camping, rock climbing, fishing, hunting, unstructured playing, climbing trees, building dens, and walking (Tseng and Wang, 2020; Cont et al., 2023; van Heel et al., 2024). Material connection, on the other hand, refers to how humans consume and utilize resources from nature (Ives et al., 2018) such as growing food for personal consumption, picking fruits, receiving organically grown vegetables, and hunting (Helne, 2022; van Heel et al., 2024). Such activities foster practical knowledge of natural systems while simultaneously providing opportunities for restorative experiences that enhance physical wellbeing and ecological understanding through sustained material engagement with the natural world. It is important to distinguish this material engagement, referring to tangible, physical interactions with nature, from materialistic and utilitarian values (e.g., centered on wealth, possessions, image and status) which have been found negatively correlated with nature connectedness (Barragan-Jason et al., 2022).

ESE delivers direct natural encounters that generate embodied knowledge particularly significant for adolescents, whose identity formation processes are enhanced through immediate sensory engagement with natural environments. These direct experiences create somatic knowledge that becomes integrated into adolescents’ developing cognitive schemas. This process may be especially salient during adolescence due to enhanced cognitive flexibility and openness to new experiences. Adolescents naturally seek out novel and challenging experiences as part of their developmental drive for exploration and autonomy, making sensory engagement with nature particularly meaningful during this period (Tseng and Wang, 2020). However, opportunities for such direct engagement with nature are not equal for everyone. Access to safe, nearby, welcoming natural environments varies across socio-economic and geographical contexts, which shapes the experiential connection with nature [European Environment Agency (EEA), 2022].

#### 4.1.4 Philosophical-ethical-spiritual connection

*Philosophical-ethical-spiritual* (PES) dimension depicts a profound connection to nature grounded in ethical, philosophical, and spiritual perspectives, emphasizing humanity’s role, responsibilities, and intrinsic and symbolic value of the world. This dimension, evident in 20 studies as seen in Table 3, includes two subdimensions: *philosophical and ethical perspectives* (PHET) and *spiritual and symbolic appreciation* (SPR). PES perspectives similarly emerge through the integration of cognitive insights and lived experiences, forming coherent worldviews that provide meaning, purpose, and ethical viewpoints for human-nature relationships. It addresses reflective and value-based components of nature connectedness, wherein people develop sophisticated understanding and appreciation of nature. The foundational worldviews and moral philosophies that shape one’s understanding of the relationship with nature, including people’s role and the ethical responsibilities toward protecting and caring for the natural environment pertain PHET. A deep, personal bond with nature combined with recognition of its esthetic and intrinsic value, involving spiritual or transcendent experiences in natural settings is captured by SPR. PES operates as a unified construct where philosophical understanding informs ethical responsibility, which is also deepened through spiritual experience, creating a transformative worldview.

PHET represents an intertwined standpoint presented as complementary aspects of a necessary shift in how humans relate to the natural world. It involves people’s deepest ideas about what nature is and why it matters (Helne, 2022). The philosophical level is argued to be the most internal and holds the highest leverage for driving significant, systemic change toward sustainability (Francomano et al., 2022). Shifting the idea from the traditional dichotomous separation of humans and nature to a relational perspective, “non-dualist turn,” challenges anthropocentric worldviews by positing that humans are inextricably intertwined with nature (Helne, 2022). Philosophical dimension is, also called NR-Perspective by Nisbet et al. (2009), articulated as nature-oriented worldviews that affect individuals’ perception of personal responsibility regarding how human behavior influences all forms of life, representing deeper philosophical positions about humanity’s role within ecological networks. These philosophical stances are closely linked with ethical perspective that offer moral direction for human conduct toward nature by challenging human domination, rejecting the exploitation of nature, and criticizing unsustainable consumption choices. The combination of philosophical and ethical elements within nature connection demonstrates the understanding that human-nature bonds are not simply experiential or affective but essentially involve moral and conceptual deliberation in an interdependent world.

SPR embodies a dimension that shows profound symbolic meaning-making, and transcendent connection between humans and the natural world. For example, the sentiment expressed in “the dark clouds and dark thoughts disappear and the light wins” shows how spiritual connection facilitates psychological healing and symbolic meaning making, by demonstrating nature’s capacity for inner transformation (Helne, 2022, p. 538). However, significant cultural variation exists in how spirituality manifests within nature connectedness. Students in the United States, for example, revealed individual variation in spiritual expression with some describing “deeply spiritual connection to nature”, while others mentioned that highly connected individuals might lack religious beliefs (Duke and Holt, 2023, p. 9). On the other hand, adolescents in Taiwan did not understand the concept of spirituality and could not express spiritual experiences in nature (Tseng and Wang, 2020). This contrast markedly with Indigenous perspectives where spirituality represents a central component of holistic wellbeing, exampled by Australian Aboriginal understanding that ill-health extends beyond physical symptoms to the expression of deeper issues such as spiritual and emotional disconnection from the land (Mikhailovich and Pavli, 2011). Such cultural variations suggests that nature connectedness maybe deeply embedded within specific worldviews and traditions that shape how individuals interpret and experience their relationship with the natural world.

PES operates as a constitutive dimension in which philosophical understanding informs ethical responsibility and is deepened through spiritual and symbolic appreciation, together shaping a worldview of human-nature relationships. As adolescence is marked by the consolidation of personal values, emerging worldviews, and increased capacity for abstract and reflective meaning-making (Tseng and Wang, 2020), PES becomes significant during this developmental stage. As adolescents form identities, they begin to locate themselves within a broader moral and existential narratives, including questions about humanity’s place in nature and responsibilities toward it. In this context, philosophical and ethical perspectives may develop not only through cognitive understanding but also through emotionally intense experiences such as grief in response to environmental destruction (Karami and Gorzynski, 2022) or heartbreak when witnessing environmental damage (Tseng and Wang, 2020). Spiritual and symbolic appreciation may similarly become more prominent as adolescents interpret nature as a source of meaning (Helne, 2022), allowing PES to function as a value-based dimension of nature connectedness.

#### 4.1.5 Sustainable practices and stewardship

*Sustainable practices and stewardship* (SPS) constitutes a commitment to environmental sustainability demonstrated through responsible behaviors, lifestyle choices, and a dedicated effort to care for and protect natural spaces. In this framework, SPS is conceptualized as a constitutive dimension of nature connectedness, reflecting internalized sustainability-oriented values and stewardship commitment rather than being treated solely as a behavioral outcome. This dimension, evident in nine studies, includes two subdimensions: *sustainable practices* (SUS) *and stewardship commitment* (STW). Environmentally friendly behaviors such as recycling, reducing waste, and environmentally conscious decisions are represented by SUS. The sense of duty and active commitment to protect, respect, and care for natural environments and local green spaces signifies the STW.

SUS is fundamentally connected to nurturing meaningful human-nature relationships. Being connected with nature is associated with pro-environmental attitudes and a willingness to engage in pro-environmental behaviors. Individuals who feel connected to nature are more likely to exhibit pro-environmental attitudes and readiness to participate sustainable actions (Grimwood et al., 2018). However, the pathway from nature connectedness to sustainable practices might not be straightforward, as structural barriers including sociocultural factors and even limited financial means might hinder these efforts (Galli et al., 2017; Helne, 2022). Sociocultural factors such as conflicting neighborhood relationships, high crime rates, and poorly maintained public spaces can also prevent positive engagement outdoor and care for nature (Galli et al., 2017). Therefore, interventions should aim to cultivate wellbeing through everyday experiences with nearby nature, fostering community connection, and providing accessible and safe green spaces.

STW is intrinsically linked to a deep connection to nature, as it forms the basis for a pro-environmental citizenry (Grimwood et al., 2018). People who are more connected to nature are more likely to actively protect it (Duke and Holt, 2023). Stewardship refers to a commitment to action to protect nature, stemming from a place of caring and having a connection to the sources that an individual wants to protect. The willingness to act can manifest in various ways such as seeking education, establishing career goals, volunteering, donating money, reducing one’s footprint, or acting politically (Duke and Holt, 2022), or in simpler ways such as switching off unnecessary lights, reusing, reducing, and recycling (Mayer and Frantz, 2004). This commitment can also be understood as part of a developing environmental identity that acts as a compelling motivator for heightened environmental awareness across social, political, and personal spheres, driving conservation-oriented behaviors (Duke and Holt, 2023). Essential to meaningful environmental stewardship is recognizing humanity’s interconnectedness with nature rather than viewing one’s self as distinct from it (Helne, 2022).

SPS embodies a synthesis of nature connectedness where cognitive understanding, embodied knowledge, emotional investment, and philosophical commitment translate into tangible environmental behaviors and stewardship actions. This form represents a transformation where people move beyond passive appreciation to active environmental engagement. The conceptualization here aligns with the Nature-based Literacy Framework’s recognition that stewardship represents the culmination of interconnected developmental processes rather than isolated outcome (Barrette et al., 2025). This culminative process may be salient during adolescence, when identity formation accelerates and adolescents increasingly define themselves through socially visible values and practices (Branje, 2021). In this context, stewardship becomes and identity-relevant mode of participation.

#### 4.1.6 Psychological and physical wellbeing

In our conceptual framework, *psychological and physical wellbeing* (PPW) captures the wellbeing-related psychological and embodied states through which nature connectedness is felt, maintained, and given personal meaning. This dimension, evident in three studies as seen in Table 3, includes two subdimensions: *psychological wellbeing* (PSY) and *physical wellbeing* (PHY). These two terms can be considered conceptually inseparable with the dynamic interplay between both psychological and physical subdimensions impacting nature connectedness with the bidirectional relationship of each subdimension influencing the other. We included PPW as dimension into the framework, because the relationship between nature connection and wellbeing is increasingly recognized as reciprocal (Gal and Domotor, 2023).

Within our comprehensive review, a small subset of studies (*n* = 3) explicitly positioned wellbeing-related constructs within broader conceptualizations of nature connectedness. Two quantitative studies explicitly operationalized wellbeing as an integral component of the construct. Mikolajczak et al. (2023) incorporated psychological wellbeing derived from nature within their measure of emotional connection to nature, specifically in the LCNR scale that captured feelings of love, beauty, joy, and wellbeing experienced in natural settings. Tseng and Wang (2020) identified psychological restoration including refreshment, relief, and stress reduction, in their three-construct model of adolescent nature connectedness based on phenomenological interviews. Additionally, Grimwood et al. (2018) documented through instructor narratives in urban outdoor education programs that resilience developed hand-in hand with children’s nature connectedness, suggesting these dimensions may be experientially intertwined. Although limited in number, these studies indicate that wellbeing has been conceptualized as part of nature connectedness, supporting our inclusion of PPW as a constitutive dimension. By contrast, much of the wider literature conceptualizes psychological wellbeing and health as consequences or correlates of nature connectedness (Gal and Domotor, 2023). This divergence highlights an important conceptual question regarding whether wellbeing is external to nature connectedness or constitutive of how connection is experienced.

We propose that PPW can be meaningfully treated as a constitutive dimension of nature connectedness when wellbeing is understood not merely as an outcome, but as part of how connection is experienced. This approach is empirically grounded in how people subjectively experience their relationship with nature. In Tseng and Wang (2020) study, adolescents described their most connected moments in nature as inherently including feelings of restoration and wellbeing, suggesting that connection and wellbeing experienced as unified rather than sequential. Similarly, in Martyn and Brymer (2016) study, healthy perspective including wellbeing was one of the themes emerged based on people’s reflections on what being in nature meant to them. Alongside this, the operationalization of wellbeing within emotional connection measures (Mikolajczak et al., 2023) implies these constructs may be difficult to disentangle in both subjective experience and measurement. When people cannot separate feelings of wellbeing from the feelings of connection in their nature experiences, and when researchers operationally measure wellbeing as a component of connection rather than separate construct, the distinction between constitutive and consequential becomes less meaningful. Our framework reflects this empirical reality while acknowledging that wellbeing’s role in nature connectedness remains an area of ongoing theoretical development.

People with greater nature connection show higher levels of psychological wellbeing, particularly a strong sense of meaning (Nisbet et al., 2011) which may be fostered through a profound connection with nature, as people perceive their existence as part of a larger, interconnected whole (Howell et al., 2011). This reciprocal pattern further illustrated by the positive feedback loop described by Nisbet et al. (2011), whereby people who feel connected to nature are more likely to engage in pro-environmental behaviors, which in turn enhance their wellbeing and connection to nature. As their wellbeing improves, so does their sense of connection, reinforcing the cycle demonstrating how wellbeing functions as a part of connection itself.

Epidemiological evidence indicates that high prevalence of mental illnesses during adolescence in Australia and globally. Every one in seven adolescents have experienced a mental illness (Australian Institute of Health and Welfare [AIHW], 2025). However, there are modifiable lifestyle factors having considerable influence on mental wellbeing throughout this critical developmental period (Australian Institute of Health and Welfare [AIHW], 2025; Smout et al., 2023). Despite established public health guidelines published on the 2022 Australian Report Card on Physical Activity for Children and Youth, the majority of adolescents fail to meet behavioral benchmarks, for instance, as of 2022, recent data show that approximately 90% Australian youth exceed the recommended limit of 2 h of recreational screen time per day (Smout et al., 2023). This observation is particularly concerning given emerging research revealing a negative influence of screen time on time spent in nature (Minor et al., 2023) and decreased time in nature is associated with diminished mental health. Indeed, increased engagement with natural environments shows strong associations with improved wellbeing (Gal and Domotor, 2023). Furthermore, nature-based activities frequently involve physical movement, thereby offering dual health benefits through the benefits of physical activity.

#### 4.1.7 Dimensional interrelationships

The Integrated Nature Connectedness Framework is a system comprising distinct yet interconnected dimensions. This system approach is based on the proposition of a dynamic relationship that is suited to the complexities of human-nature relationships. CC and ESE serve as the mutually influential foundational dimensions for adolescent nature connectedness development. From these foundational dimensions, more complex psychological processes emerge that transform basic knowledge into personally meaningful relationships: ERC and PES perspectives represent developmental process that emerge from foundational dimensions while showing reciprocal influence on both foundational dimensions and each other, particularly relevant during adolescence when identity formation and value development are central. The interaction of these dimensions manifests in tangible behaviors that both express and reinforce nature connectedness. Therefore, SPS represents the action dimension where all foundational and developmental dimensions converge into behavioral expression while reinforcing and deepening connections across other dimensions. Then, PPW holds a place as an emergent outcome arising from the harmonious integration and mutual reinforcement of all other dimensions, while simultaneously functioning as an enabling condition that facilitates continued engagement and development across the framework. This dual role exemplifies the reciprocal architecture of this dimension, where wellbeing both results from subjective nature connection and creates psychological and physical capacity for deeper engagement with the natural world. The emergent properties of wellbeing which includes stress reduction, enhanced mood regulation, improved cognitive function, and strengthened sense of purpose establish self-reinforcing cycles that sustain motivation for continued connection with nature. This provides adolescents essential resources for navigating developmental challenges and amplifying the effectiveness of all other dimensions.

### 4.2 Limitations and future research directions

In order to contextualize the results appropriately, it is necessary to recognize the study’s inherent limitations. Although this study employed comprehensive search strategies across several databases, employing search criteria limited to specific terms related to NC may potentially cause to exclude studies that used alternative terminology. Expanding search parameters to include terms such as “human-nature relationships” or “environmental identity” may have produced additional relevant literature, however, such expansion would have made this study methodologically unmanageable and beyond the reasonable scope. The literature search was also limited to English-language publications within a specific timeframe, which excludes potentially valuable studies published in other languages and beyond the defined time period, thereby introducing the cultural and linguistic bias into the findings.

A key limitation of the present framework concerns cultural scope and external validity. Although the manuscript occasionally draws on Indigenous or relational epistemologies, the evidence base informing the framework is predominantly derived from Westernized, school-based research. Accordingly, the proposed framework is likely to be most applicable to urban, schooled adolescents, and its transferability to Indigenous and non-Western contexts should not be assumed. Future research should test the framework through culturally grounded validation and context specific adaptation. Future research should also prioritize the development of robust assessment instruments that capture the dimensional complexity of INCF. Empirical validation across diverse cultural and developmental contexts is essential to establish the framework’s generalizability and to explore potential variations in nature connectedness. Longitudinal and intervention studies could further explore different patterns, elucidating how various dimensions of NC develop or respond to targeted educational programs. Investigations into innovative pedagogical approaches that align with the framework’s conceptual foundation could inform evidence-based interventions aimed at facilitating connection to nature.

The INCF provides a structured foundation for systematic investigation of nature connectedness across diverse populations and contexts. Its multidimensional structure enables researchers to examine differential patterns of connection. The framework also offers significant methodological advantages through its multifaceted applicability across diverse research approaches. The framework’s dimensional structure also provides a foundation for developing assessment tools such as interview protocols, observational frameworks, and survey instruments that capture the multifaceted complexity of human-nature relationships. Furthermore, educators can utilize the framework to develop targeted activities that strengthen particular dimensions, while practitioners can assess individual or group profiles across the six dimensions to tailor approaches accordingly.

## 5 Conclusion

This study proposes the Integrative Nature Connectedness Framework (see Table 4) that integrates fragmented conceptual perspectives to address the multifaceted complexity of human-nature relationships. The framework challenges unidimensional approaches by organizing nature connectedness into six core dimensions, providing a comprehensive foundation for understanding these psychological interactions. This integrative structure offers a developmentally sensitive basis for examining adolescents’ nature connectedness. The framework represents a significant conceptual advancement by synthesizing disparate frameworks that have previously operated in isolation. It offers a unified paradigm through which the multifaceted nature of human-nature relationships can be systematically explored and understood. With its clarifying multidimensional structure the Integrative Nature Connectedness Framework provides a basis for measurement approaches that can better distinguish adolescents’ connection to nature from that of children and adults, thereby improving the precision of future research and practice during this pivotal time of life.

## Glossary

NC: nature connectedness
INCF: Integrated Nature Connectedness Framework
ERC: Emotional and Relational Connection
CC: Cognitive Connection
ESE: Experiential and Sensory Engagement
PES: Philosophical-Ethical-Spiritual Connection
SPS: Sustainable Practices and Stewardship
PPW: Psychological and Physical Wellbeing
EMO: Emotional Attachment
INT: Integration with Nature
COG: Cognitive Attributes
KNW: Knowledge of Natural Places
SOM: Somatic Connection
MARE: Material-recreational Engagement
PHET: Philosophical and Ethical Perspectives
SPR: Spirituality and symbolic appreciation
SUS: Sustainable Practices
STW: Stewardship Commitment
PSY: Psychological Wellbeing
PHY: Physical Wellbeing
