# Supplementary material for: Nature connectedness framework for adolescents: an integrative review
Source: Front Psychol. 2026 Mar 4;17:1721907. doi: 10.3389/fpsyg.2026.1721907 (PMC12995616; doi:10.3389/fpsyg.2026.1721907)
Supplement: Supplementary file 1 [file Table_1.DOCX]

**Supplementary Materials**

Table: Themes and constructs

| themes (frequency) | wellbeing (18) | environmentally friendly behaviours (15) | human-nature interactions (14) | educational practices (12) | environmental sensitivity and perception (8) | self-concept and identity (5) | personal values and traits (5) |
| --- | --- | --- | --- | --- | --- | --- | --- |
| construct (frequency) | - wellbeing, (14) - mental health, (3) - lower anxiety, (1) | - pro-environmental behaviours, (5) - pro-conservation behaviours, (1) - organizational behaviour, (1) - pro-environmental attitudes and behaviours, (1) - green purchase intentions, (1) - healthy food attitudes and behaviours, (1) - food leftover reduction intention, (1) - environmental stewardship, (1) - conservation attitudes, (1) - dietary choices, (1) - commitment to engage in environmentally responsible behaviours, (1) | - urban nature experience, (2) - contact with nature, (2) - human-nature interactions, (1) - authentic situations, (1) - marine wildlife experiences, (1) - places of nature contact, (1) - sensory experiences, (1) - nature-based recreation, (1) - exposure to natural environments, (1) - childhood and adult nature experiences, (1) - interacting with nature (intentional, incidental, and indirect), (1) - nature-based therapy, (1) | - environmental citizen science, (2) - a college level ecology course, (1) - urban outdoor educational practices - nature based learning, (1) - experiences in kitchen garden program, (1) - nature nursery, (1) - project-based learning, (1) - forest school training, (1) - outdoor adventures, (1) - educational practices, (1) - residential outdoor trips, (1) - wilderness expedition, (1) | - eco-centric perspective, (1) - climate change attitudes, (1) - perception of nature, (1) - environmental consciousness, (1) - soundscape perception, (1) - perceptions of climate change, (1) - biospheric values, (1) - environmental noticing, (1) | - self-esteem, (1) - science identity, (1) - interdependent self-construal, (1) - self-identity, (1) - confidence, (1) | - empathy, (1) - empathic tendency, (1) - perspective taking, (1) - personal values, (1) - characteristic strength, (1) |
